# Rice bran extract attenuates cognitive impairment by enhancing pancreatic β-cell insulin secretion in STZ-induced diabetic rats targeting the PPARγ/PDX1 pathway

**DOI:** 10.1007/s11011-025-01639-1

**Published:** 2025-06-19

**Authors:** Madonna Magdy Youssef, Mohammed Farrag El-Yamany, Reham Mahmoud Abdel-Kader, Ola Ahmed Heikal

**Affiliations:** 1 Pharmacovigilance Department, Egyptian Drug Authority, Giza, Egypt; 2 Pharmacology & Toxicology Department, Faculty of Pharmacy, Cairo University, Cairo, Egypt; 3 Pharmacology & Toxicology Department, Faculty of Pharmacy and Biotechnology, German University, Cairo, Egypt; 4 Narcotics, Ergogenics & Toxins Department, National Research Center, Giza, Egypt

**Keywords:** Insulin, Diabetes, Rice bran extract, PPARγ, Cognition

## Abstract

Type I diabetes (T1D), also known as juvenile diabetes, is an autoimmune disease that causes gradual destruction of pancreatic cells and leads to intellectual disability, neuropathy, cognitive impairment, and impaired learning ability in children. Despite standard treatment with synthetic human insulin, T1D patients can maintain up to 40% of their insulin-producing islets. PPARγ receptor activation research that aims to restore β-cell biology could help reverse the loss of pancreatic mass that comes with getting older and improve β-cell function. Egyptian RB ethanol extract (RBE), previously reported with PPARγ agonist activity, showed an increase in insulin secretion both in vivo and in INS-1 cells. The exact antidiabetic RBE mechanism is still unclear. The present study aims to investigate the molecular RBE mechanism in glucose-stimulating insulin secretion and restoration of β cell function. A diabetic rat streptozotocin (STZ) model was used; five groups were designed. The STZ-diabetic rats were treated with RBE daily for 21 days compared to an insulin-treated group. Biochemical parameters and quantitative RT-PCR of β-cell genes related to the PPAR/PDX1 signaling pathway were performed, and the influence on cognitive ability was confirmed by behavioral testing (Y-maze and NOR) and histological examination. The RBE-treated group reversed blood glucose, Glut2, Ca2 +, and insulin levels in diabetic rats, with pancreatic insulin levels significantly increasing compared to the insulin group. With the exception of PDX1, RBE boosted PPARγ, SERCA, and PrKC gene expression. RBE also restored cognitive functions. This study suggests that RBE may enhance memory and cognition by increasing peripheral insulin secretion through PPARγ regulator activity.

**Graphical abstract:**

Schematic pathway for insulin secretion via PPAR-γ dependent pathway in type 1 diabetes

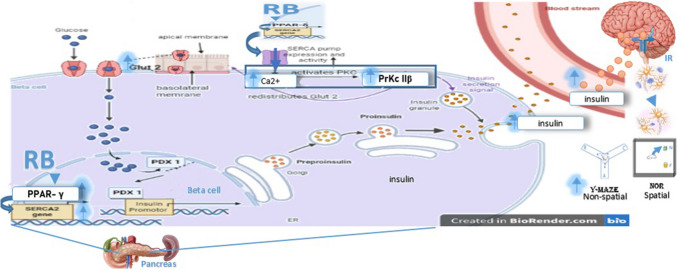

**Supplementary Information:**

The online version contains supplementary material available at 10.1007/s11011-025-01639-1.

## Highlights

Glucose transport in β-cells plays a significant role for insulin secretion.

Egyptian Rice bran extract treatment enhanced insulin secretion.

Egyptian Rice bran extract boosted PPARℽ, SERCA, and PrKC gene expression.

Egyptian Rice bran extract restored pancreatic structure more effectively.

RBE may enhance memory and cognition by increasing peripheral insulin secretion.

**Supplementary Information:**

The online version contains supplementary material available at 10.1007/s11011-025-01639-1.

## Introduction

*Diabetes mellitus* is a prevalent chronic illness globally. Type 1 diabetes (T1D) is a long-term, organ-specific autoimmune disorder that destroys pancreatic β-cells gradually and is known as juvenile diabetes (age > 20). *Diabetes mellitus* is the seventh most common cause of death in the United States (Sapra and Bhandari 2020; Leslie et al. 2021; Kim et al. 2021a, b). In 2021, the International Diabetes Federation (IDF) reported 1.2 million cases of children and adolescents with T1D worldwide, with an expected annual increase of 184,100 cases and prevalence of 2% in Africa and 10% of cases in Egypt (IDF 2021a, b).

Anomalous glucose and insulin levels in childhood T1D patients can lead to mental impairment and neuropathy (Jin et al. 2022; Li et al. 2021; Xu et al. 2021). This is a common microvascular diabetes complication (Galiero et al. 2023) resulting in cognitive disturbances and limited learning ability in children (Lacy et al. 2022) with a significant medical and financial burden (IDF 2021a, b).

Typical treatment for T1DM is synthetic human insulin. However, its administration has numerous negative consequences in all age groups, where severe uncontrollable hypoglycemic events negatively impact cognitive and neuronal function (Hultström et al. 2014; Jin et al. 2022).

It is believed that T1D patients almost completely lose β-cells at the onset of the disease. Further research implies a functional deficit rather than absolute β-cell loss, which may preserve up to 40% of insulin-producing islets and restore insulin production (Coppieters et al. 2012; Krogvold et al. 2015a; Leete et al. 2016). Also, isolated islets from T1D patients were reported to restore insulin production in a non-diabetic environment (Krogvold et al. 2015b). Improving β-cell biology is a promising research area for T1D treatment (Atkinson et al. 2019; Cobo—Vuilleumier et al. 2018). Activation of PPARγ (Peroxisome Proliferator-Activated Receptor), a transcriptional regulator involved in glucose metabolism, and its synthetic agonists as thiazolidinedione (TZD family) have been reported to maintain β-cell proliferation and reverse the age-related reduction in pancreatic mass in both rats and mice (Shimabukuro et al. 1998; Higa et al. 1999; Finegood et al. 2001). TZDs have been reported to enhance insulin secretion by controlling several key β-cell genes that potentiate glucose-stimulating insulin secretion (GSIS) (Evans-Molina et al. 2009; Moibi et al. 2007). However, TZDs are not recommended as first-line medication due to their numerous negative consequences, including edema, heart failure, liver damage, and bladder cancer (Eggleton and Jialal 2020; Hwang et al. 2021).

Thus, the proven use of some traditional medicinal plant products as “supportive therapy” for improving glycemic abnormalities in individuals with type 1 and type 2 diabetes was reported. Their use provided an alternative approach to alleviate hyperglycemia with a safe, practical, cost-effective, and convenient way to treat diabetic complications (Rahman et al. 2022).

Rice bran, a product of rice milling, is used in traditional medicine to treat various health disorders, supported by experimental and clinical evidence (Ahuja et al. 2008; Umadevi et al. 2012). According to in vitro research, RB can reduce hyperglycemia by increasing glucose uptake in 3 T3-L1 adipocytes, triggering the expression of glucose transporters, and blocking the activity of α-glucosidase and α-amylase (Boue et al. 2016; Wahyuni et al. 2016). RB oil is commonly used for cooking in Thailand and India. A blend of 80% RB oil and 20% sesame oil found significant reductions in fasting blood glucose, postprandial glucose, and glycated hemoglobin levels (Devarajan et al. 2016).

One of the documented rice bran products with PPARγ agonistic activity is the standardized ethanolic RBE (Abd El Fattah et al. 2020; El-Din et al. 2021; Mostafa et al. 2018). RBE is one of the innovative natural products of the Egyptian rice bran and holds several health benefits (Bhatia et al. 2016: Jolfaie et al. 2016: Kaup et al. 2012: Meselhy et al. 2018: Shendy et al. 2024). Kaup et al. reported a RBE antidiabetic effect through a concentration-dependent increase in insulin levels in INS-1 cells and in vivo in plasma (Kaup et al. 2012). RBE comprises a splendid set of active phytochemical components, including ℽ oryzanol, tocopherols, tocotrienols, policosanol, and polyunsaturated fatty acids (Al-Okbi et al. 2014; Kalita et al. 2022).

However, the exact underlying mechanism of RBE for the increased insulin release remains unclear and needs more investigation. In view of RBE's documented agonistic effect on PPARγ (Abd El Fattah et al. 2020; El-Din et al. 2021; Mostafa et al. 2018), we propose a possible mechanism for RBE to restore β-cell function via PPARγ regulation.

In this study, we investigated the effect of RBE on the PPARγ/PDX1 pathway, suggesting that RBE PPARγ agonistic activation would promote the sarcoendoplasmic reticulum calcium ATPase pump (SERCA2) to transfer Ca^2+^ molecules across the ER membrane. This maintains high Ca^2+^ levels within the ER lumen. The suggested increase in Ca^2+^ subsequently would activate protein kinase C (PrKC βII) (Kono et al. 2012; Trexler and Taraska, 2017). PrKC βII redistributes glucose transporter GLUT2, transporting glucose molecules inside the β-cell (Cohen et al. 2014). This in turn activates PDX1, a transcriptional regulator that controls insulin gene expression (Xia et al. 2014; Zhou et al. 2014).

Therefore, the PPARγ-targeted activation approach in insulin secretion raised our concern about how RBE would affect β-cell function and insulin secretion. Given the recently reported RBE-PPARγ agonist activity and the documented glycemic control effect of RB products and their constituents, we focused on investigating the RBE underlying mechanism. This was done in the diabetic rat STZ model compared to exogenous insulin.

## Material and methods

### Animals

Male Sprague–Dawley rats weighing 180 to 230 g were used in the study. Male rats were chosen to ensure a reliable and reproducible diabetic model with consistent metabolic outcomes, as they were reported to demonstrate a more robust and stable hyperglycemic response following STZ administration (Furman 2015; Gurley et al. 2006; Rocha et al. 2023). They were obtained from the central administration animal colony (National Organization for Drug Control and Research, Giza, Egypt) and maintained with a standard laboratory diet and water ad libitum. Animals were maintained in a temperature- and pressure-controlled room (23–24 °C and 40–60% relative humidity) and exposed to 12-h dark and light cycles. Before being used in the experiment, the animals went through an acclimatization phase.

### Adherence to the ethical standard

The Research Ethics Committee at Cairo University (Cairo, Egypt, PT 3080) approved this research methodology. The guidelines for the care and use of laboratory animals, issued by the United States National Institute of Health (NIH publication No. 85–23, revised 1996), strictly governed the handling of animals and all procedures (Clark et al. 1997).

### Chemicals

Stabilized RBE was acquired from Health Tech Company at 87 Ramsis Street, Cairo, Egypt. A RBE dose of 100 mg/kg/day, chosen based on previous literature that showed efficacy and no toxicity (Abd El Fattah et al. 2020; El-Din et al. 2021; Heikal et al. 2015; Mostafa et al. 2018; Shendy et al. 2024), was administered orally dissolved in 0.4% DMSO (Mostafa et al. 2018). Long-acting insulin (100 UI/mL Insulin Lantus) was acquired from Sanofi-Aventis, France, and administered via a daily subcutaneous (SC) injection at a dose of 5 IU/200 g of body weight/day (Luippold et al. 2016; Schaschkow et al. 2016). STZ was acquired from Sigma-Aldrich, Germany, and administered via an intraperitoneal injection (IP) for 4 days at a dose of 20 mg/kg of body weight per day.

### Preparation of stabilized RBE

Initially, the lipase enzyme in rice bran was inactivated after milling by the high-temperature short-term method (HTST) to produce stabilized rice bran. Then, for ethanol extraction, rice bran powder was mixed with 95% alcohol at a ratio of 1:3 and then macerated overnight at 50 °C for three consecutive times. The extract was vacuum-evaporated at a temperature not higher than 50 °C. The phytochemical profile of RBE was identified as previously published (supplementary data S2-S5). The content of γ-oryzanol and vitamin E was identified using HPLC analysis (Agilent 1100). Fatty acid methyl esters analysis was performed by GLC using the Agilent GC system. The analysis of the polyphenolic content of RBE was measured using liquid chromatography-electrospray ionization tandem mass spectrometry (LC–ESI–MS/MS) with an Exion LC AC system for separation and a SCIEX Triple Quad 5500 + MS/MS system equipped using electrospray ionization (ESI) for detection. The oily extract was kept in the refrigerator and warmed immediately before use to 37 °C in a water bath.

### Diabetes induction

Type 1 diabetes was induced by intraperitoneal injection of multiple low doses (20 mg/kg for four consecutive days) of freshly prepared Streptozotocin (STZ) dissolved in 0.1 M citrate buffer (pH 4.5) after an overnight fast, given only a 5% glucose solution to prevent initial hypoglycaemic mortality (Youssef et al. 2020). Diabetes was confirmed by drawing blood from the retro-orbital vein and assessing blood glucose level using a colorimetric assay kit (Cat. No. GL 1320, Biodiagnostic, Cairo, Egypt). Animals displaying fasting blood glucose levels greater than 450 mg/dl were used in the present study. Worth mentioning is that in this period (4 days), control groups received citrate buffer I.P.

### Experimental design

We used sixty rats (180–230 g) in our research study. Animals were divided into five groups, each comprising twelve rats (5, 12). According to Fig S1, after the first four days of diabetes induction, for 3 weeks (the experimental duration), the negative control group (CON) (*n* = 12) received 0.4% DMSO orally, and the RB-treated control group (CON + RB) (*n* = 12) received 100 mg/kg/day of RBE dissolved in 0.4% DMSO orally (Mostafa et al. 2018). DMSO's selected concentration adheres to established guidelines (< 1% v/v solutions for in vivo injections, Galvao et al. 2014) and is within the safe range reported in previous studies that assess neuro-experimental models (Mostafa et al. 2018; Abd El Fattah et al. 2020; El-Din et al. 2021; Shendy et al. 2024). The 0.4% DMSO was given to negative control (untreated non-diabetic) and positive control (treated non-diabetic) groups to ensure that any observed behavioral or neurophysiological changes can be attributed to RB extract rather than to DMSO exposure.

Diabetic animals (*n* = 36) were further randomly assigned into 3 groups. The RB-diabetic treated group (Diabetic + RB) (*n* = 12) received 100 mg/kg/day RBE dissolved in 0.4% DMSO orally (Mostafa et al. 2018), and the insulin-diabetic treated group (*n* = 12) received 5 IU/200 g of body weight/day INS subcutaneously (Luippold et al. 2016; Schaschkow et al. 2016). Diabetic control groups were administered 0.9% saline throughout the time of the experiment. It was considered ethically necessary to provide the control untreated diabetic group with 0.9% saline instead of in 0.4% DMSO to maintain the untreated diabetic animals’ hydration status since STZ leads to significant osmotic diuresis and fluid loss (Anwana and Garland 1991; Graham et al. 2011; Gvazava et al. 2020). Weekly, blood glucose levels were determined using a glucose enzymatic colorimetric assay kit (Cat. No. GL 13 20, Biodiagnostic, Cairo, Egypt), and blood insulin levels were measured using an INS rat ELISA kit (Sunlong, SL0373Ra, China).

Behavioral tests were carried out for all rats under the same conditions to assess their cognitive ability. The Novel Object Recognition (NOR) test was performed 24 h after the last treatment (day 26) as previously reported (Mostafa et al. 2018; Shendy et al. 2024; Venkat et al. 2020). Then followed by Y-maze test a day later, allowing sufficient recovery time while maintaining behavioral stability (El-Din et al. 2021; Shendy et al. 2024; Bigelow et al. 2023; Melnikova et al. 2006; Paylor et al. 2006). At that point, all rats in all groups were sacrificed by cervical dislocation, and then the pancreas and the brain were excised. Three rats per group had their brains and pancreas preserved in 10% formalin for histopathological analysis. The remaining rats in each group had their pancreas divided into two pieces and stored in Eppendorf tubes, one for biochemical analysis and the other for gene expression analysis. For biochemical analysis and gene expression analysis, all Eppendorf tubes were kept at −80 °C until analysis.

### Biochemical analysis

#### Estimation of glucose level in blood

The glucose level in the blood was determined using a colorimetric assay kit (Cat. No. GL 1320, Biodiagnostic, Cairo, Egypt) according to the method of Trinder (1969). Following the manufacturer's instructions, the amount of glucose present was estimated as absorbance O.D. and measured at 405 nm using a single-beam spectrophotometer (Shimadzu UV-2401PC).

#### Estimation of calcium level in pancreatic tissue

Pancreatic calcium level content was determined by a colorimetric assay kit (Cat. No. GL 13 20, Biodiagnostic, Cairo, Egypt) according to the Gindler and King method (Gindler and King 1972). The manufacturer's instructions were followed to detect calcium quantity existing at absorbance O.D. and measured at 585 nm using a single-beam spectrophotometer {Shimadzu UV-2401PC}.

#### Estimation of insulin level (INS) in blood/pancreatic tissue

Quantitative measurements of INS levels in blood samples and pancreatic tissue were assayed using an INS rat ELISA kit (Sunlong, SL0373Ra). Appropriate assay procedures were followed according to the manufacturer's instructions. Insulin levels were estimated as absorbance O.D. within 15 min and measured at 450 nm using a titer plate reader (BioTek/SN 263732).

#### Estimation of glucose transporter 2 level (GLUT 2) in pancreatic tissue

Quantitative measurement of the GLUT 2 level was assayed using a GLUT 2 rat ELISA kit (Bioassay Technology Laboratory, E1058Ra). The manufacturer's instructions for appropriate assay procedures had been followed. Pancreatic GLUT 2 levels were assessed as absorbance O.D. within 10 min and measured at 450 nm using a titer plate reader {BioTek/SN 263732}.

#### Homeostasis model assessment of β-cell function (HOMA-β)

HOMA-β, an index of insulin secretory function, was calculated based upon the measurement of blood glucose and blood insulin concentrations according to the following equation: HOMA-beta = [blood insulin concentration (μU/ml) × 20]/[blood glucose (mmol/L)—3.5] (Reaven 2009).

### Quantitative real-time PCR gene expression of PPARγ, SERCA, PrKC, and PDX1 genes in pancreatic tissue

Total RNA was extracted from 60 rats'pancreatic tissues using the RNeasy Mini kit (Cat. No. 74101, Qiagen, Germany) following the manufacturer's instructions. The concentration and purity of the extracted RNA were determined using a nanodrop spectrophotometer (2000 c, Thermoscientific, USA). For purification of the RNA, 1500 ng/µl of the sample was added to 2 µl of wipe-out buffer (7x) and nuclease-free water to make a final volume of 14 µl. Incubate for 2 min at 42ºC in a thermocycler (BIO-RAD T100, USA). A mixture was prepared for cDNA synthesis using a QuantiTect reverse transcription kit with a 1:4:1 ratio of RT primer mix, Quantiscript RT buffer, and Quantiscript reverse transcriptase, and then added to the extracted RNA (14 µl). The samples were incubated in a thermocycler for 30 min at 42 °C and 95 °C and then stored at −80 °C until analyzed. For each sample, four genes were measured (PPARγ, SERCA, PrKC, and PDX1) using the RT2 Syber Green kit (Cat. No. 330524, Qiagen, Germany). The RT-PCR was performed using Design & Analysis Software (DA2) version 2.6.0 for Applied Biosystems QuantStudio 5 DX, involving adding 1 µl cDNA of each sample, 12.5 µl RT2 Syber Green, and 10.5 µl nuclease-free water. (Thermo Fisher Scientific, USA). Relative quantification for gene expression (RQ) was calculated by the 2-ΔΔCT equation according to Livak and Schmittgen (2001) using the β-actin reference gene for the normalizer as follows:

$$\begin{array}{l}\Delta\mathrm{Ct}\;\mathrm{control}=\mathrm{CTtarget}-\mathrm{CT}\;\mathrm{endogenous}\;\beta-\mathrm{actin}\\\Delta\mathrm{Ct}\;\mathrm{sample}=\mathrm{CTtarget}-\mathrm{CT}\;\mathrm{endogenous}\;\beta-\mathrm{actin}\\\Delta\Delta\mathrm{Ct}=\Delta\mathrm{Ct}\;\mathrm{sample}-\Delta\mathrm{Ct}\;\mathrm{control}\end{array}$$

### Histopathological examination of pancreatic tissue

Autopsy samples were taken from the pancreas of rats in all groups and fixed in 10% neutral buffer formalin for 12 h. Washing was done with tap water, and then serial dilutions of methyl alcohol (70%, 80%, 90%, 95%, and 100% consecutively) were used for dehydration. Samples were cleared in xylene for 1 h twice and embedded in soft paraffin, then hard paraffin, each for 1 h. Finally, paraffin blocks were prepared for sectioning by microtome 2–4 µ. For pancreas routine examination using a light electron microscope, the obtained tissue sections were collected on glass slides, deparaffinized, and stained with hematoxylin and eosin stain (Bancroft and Stevens 1996). Congo red staining was performed for the brains of rats in all groups using the established protocol. Formalin-fixed and paraffin-embedded tissue was cut into 6-μm-thick sections, deparaffinized, and dyed with 2.5 g of Congo red (Merck Millipore, Darmstadt, Germany) and 1 g potassium hydroxide (Merck Millipore) dissolved into 500 ml of 80% ethanol (Menter et al. 2016).

### Behavioral assessment

#### Novel object recognition test

This test assesses different interspecies aspects of non-spatial recognition memory. The main arena was a wooden, white, opaque chamber (around 40 cm × 40 cm × 60 cm). On the first day, for habituation, each rat was allowed to freely explore the arena without any objects for 10 min. For individual variability, any rat that showed abnormal exploration behavior during the habituation phase was excluded from the study. On the following day, each rat goes through a training session for visual exploration of two identical objects for 10 min, then, after a 2-h retention interval, a testing session where one of the previously explored objects is substituted with a novel object, and the animal is left 5 min to explore. In the testing session, a video was recorded of the rats'behavior. Since rodents have an innate preference for novelty, the rat with intact memory will spend more time exploring the novel object. In all sessions, each rat was removed from its home cage and placed in the middle of the open arena, and at the end, the rat was removed and placed in a holding cage. Between each rat, the apparatus was cleaned using 70% ethanol. Objects were different enough to be easily discriminated by the rat but had a similar degree of complexity (texture, shape, color patterning, and brightness) to minimize any possible induced object preference. The discrimination ratio (DR) was calculated as the time spent exploring the novel object minus the time spent exploring the familiar object divided by total exploration time (Antunes and Biala 2011; Lueptow 2017).

#### Y-maze test

This test evaluates short-term spatial memory. A three-armed, opaque wooden Y-shaped apparatus measuring 32 mm in length, 10 mm in height, and 5 cm in width and angled at a 120° angle from one another was utilized. Following an introduction to the center of the maze, the rat was given eight minutes to freely investigate each of the three arms, and the order in which it entered each arm was recorded. When its working memory is intact, the rat would prefer to explore a new section of the maze than go back to a previously explored one. The percent alternation is calculated as:

$$\left(\text{Number of alternations}/\text{number of entries}-2\right)\times 100.$$

where the number of alternations is any three successive choices of three different arms that are counted as a correct choice and the number of entries are the total possible alternations (Arai et al. 2001).

## Statistical analysis

Sample size was calculated by G*power 3.1 for 5 groups using α = 0.05, power = 0.95, and effect size = 0.6 according to previous publications (Nonaka et al. 2024, de Oliveira Fernandes et al. 2023).

All results were expressed as the mean ± S.E.M. (standard error of the mean). Following normality tests (Kolmogorov–Smirnov and Shapiro–Wilk), data were analyzed with a one-way ANOVA and then a Tukey–Kramer post-test for a one-variable multiple-group comparison. For the Y-maze, data showed non-normality in 2 groups and accordingly were analyzed with the Kruskal–Wallis test and then a Dunn's post-test. To further support the validity of our parametric analysis, we ensured equal group sizes and employed Winsorization to mitigate the influence of outliers (Glass et al. 1972; Harwell et al. 1992; Ramachandran & Tsokos 2020, Kwak and Kim 2017).

Blood glucose and blood insulin were analyzed with a two-way analysis of variance (ANOVA) followed by the Bonferroni post-test for two-variable multiple-group comparisons. In all cases, a *p*-value less than 0.05 was considered statistically significant. Statistical analysis was performed using instant automated software (GraphPad Prism Software version 9.0.1).

The F distribution formed by the variance ratios according to the degree of freedom combinations was represented by the F value to serve as a reference for the *P* value location in the data distribution (Kim 2017). Histopathological score values were considered non-parametric values, so data were analyzed by the Kruskal–Wallis test followed by a Dunn's post-test.

## Results

### Effect of rice bran extract and insulin on biochemical parameters

#### Blood glucose levels in STZ diabetic rat

**Fig. 1:**
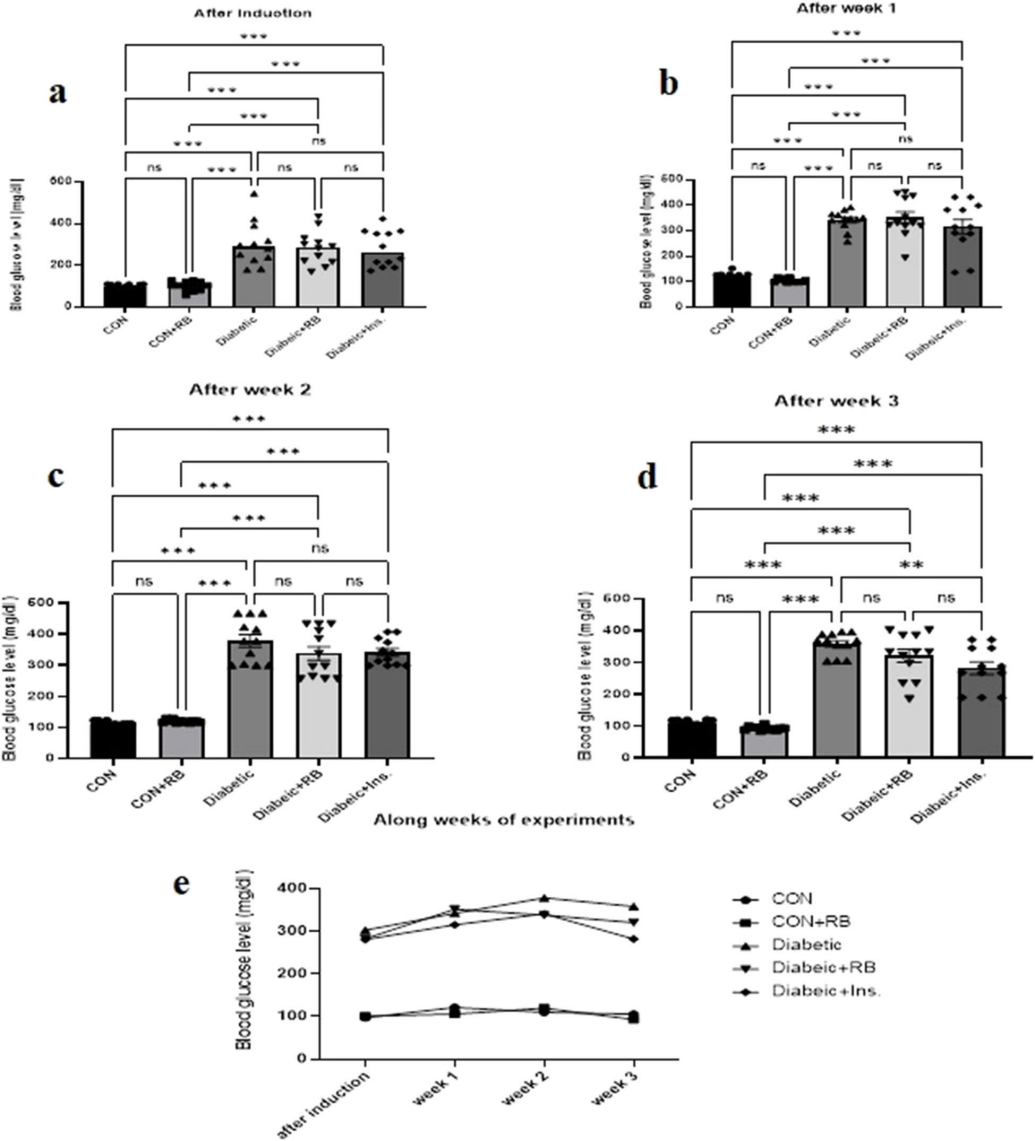
**Effect of oral treatment with RB (100 mg/kg/day) and Ins. (5 IU/200 g/day) on blood glucose level: **Effect of treatment with RB (100 mg/kg/day, P.O), and Ins. (5 IU/200 g/day, S.C)on blood glucose level **a**) after induction **b**) after week 1 **c**) after week 2 **d**) after week 3 **e**) Along weeks of the experiment in STZ diabetic rats. Rats were injected with multiple low doses of STZ on the first four days For every single week, Statistical analysis was done using One-way ANOVA followed by a Tukey post-test Along the weeks, Statistical analysis was done using two-way ANOVA followed by a Bonferroni post-test Values are presented as mean ± SEM (*n*=12)

As shown in Fig. 1, blood glucose levels differed greatly between the CON groups and the diabetic groups in all weeks of the experiment (F (220, 4) = 2481.55, *P* < 0.0001). Just after induction, before treatment, the diabetic groups showed a significant increase in blood glucose levels compared with the CON group of 189.8%. At week 3, compared to the diabetic group, blood glucose levels in the INS-treated group decreased by 21.36% significantly, and blood glucose levels in the RB-treated groups decreased by 10.62% non-significantly. There was also a significant difference between weeks within each group (F (220, 3) = 80.86, *P* < 0.0001). There was a significant decrease in blood glucose levels in both treated groups at week 3 compared to week 1 in each individual treated group.

#### Blood insulin levels in STZ diabetic rat

**Fig. 2:**
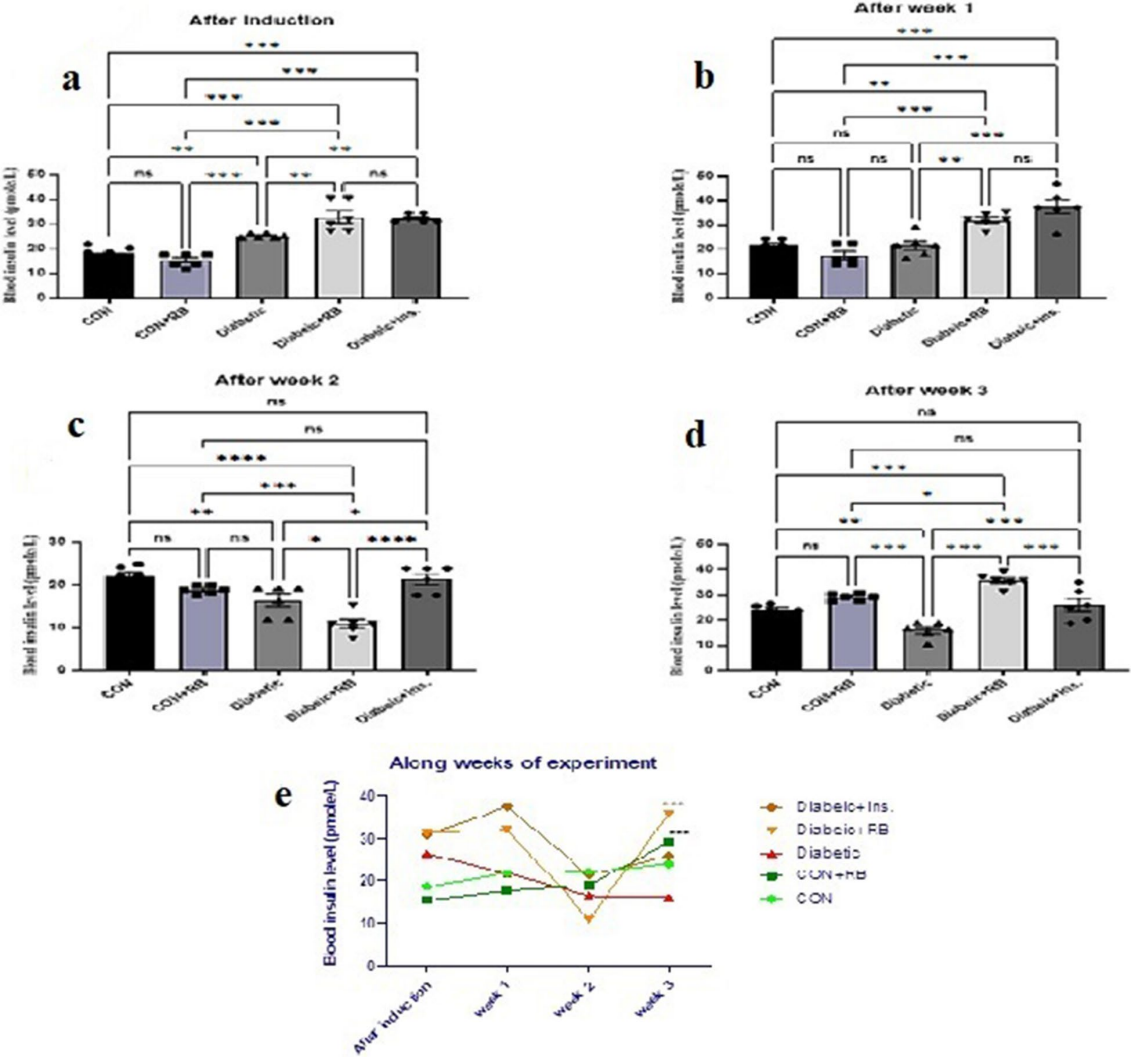
**Effect of oral treatment with RB (100 mg/kg/day) and Ins. (5 IU/200 g/day) on blood insulin level:** Effect of treatment with RB (100 mg/kg/day, P.O.), and Ins. (5 IU/200 g/day, S.C)on blood insulin level **a**) after induction **b**) after week 1 **c**) after week 2 **d**) after week 3 **e**) Along weeks of the experiment in STZ diabetic rats. Rats were injected with multiple low doses of STZ on the first four days For every single week, Statistical analysis was done using One-way ANOVA followed by a Tukey post-test Along the weeks, Statistical analysis was done using two-way ANOVA followed by a Bonferroni post-test Values are presented as mean ± SEM (*n*=6)

Throughout all weeks of the experiment, as presented in Fig. 2, the blood insulin level was highly significantly different between the various groups (F (100, 4) = 35.68). All diabetic groups displayed a significant increase in blood insulin levels after the induction by 74.119% (*P* < 0.001). At weeks 2 and 3, the diabetic group's blood insulin level decreased, showing significantly lower levels than the CON group by 26.079% (*P* = 0.0061) and 33.567% (*P* = 0.004). On the other hand, in week 3, in the treated groups, the diabetic + INS group and diabetic + RB group, insulin blood levels increased significantly by 62.15% and 122.62%, respectively, compared to the diabetic group. Interestingly, at week 3, in the CON + RB group, blood insulin level increased by 47.89%, a *P* < 0.001 significant increase from the untreated diabetic group.

As well, within each group revealed in Fig. 2e, there was a significant difference among weeks (F (220, 3) = 38.86, *P* < 0.001). At week 3, In the INS diabetic-treated group, insulin blood level showed no significant difference compared to its level in the same group at week 2. Where, in the RB diabetic treated group, there was a significant rise in insulin blood level compared to its level in the same group at week 2. Interestingly, the CON + RB group insulin blood level increased significantly at week 3 compared with week 2.

#### Effect of rice bran extract and insulin on β-cell insulin secretory function (HOMA-β)

**Fig. 3:**
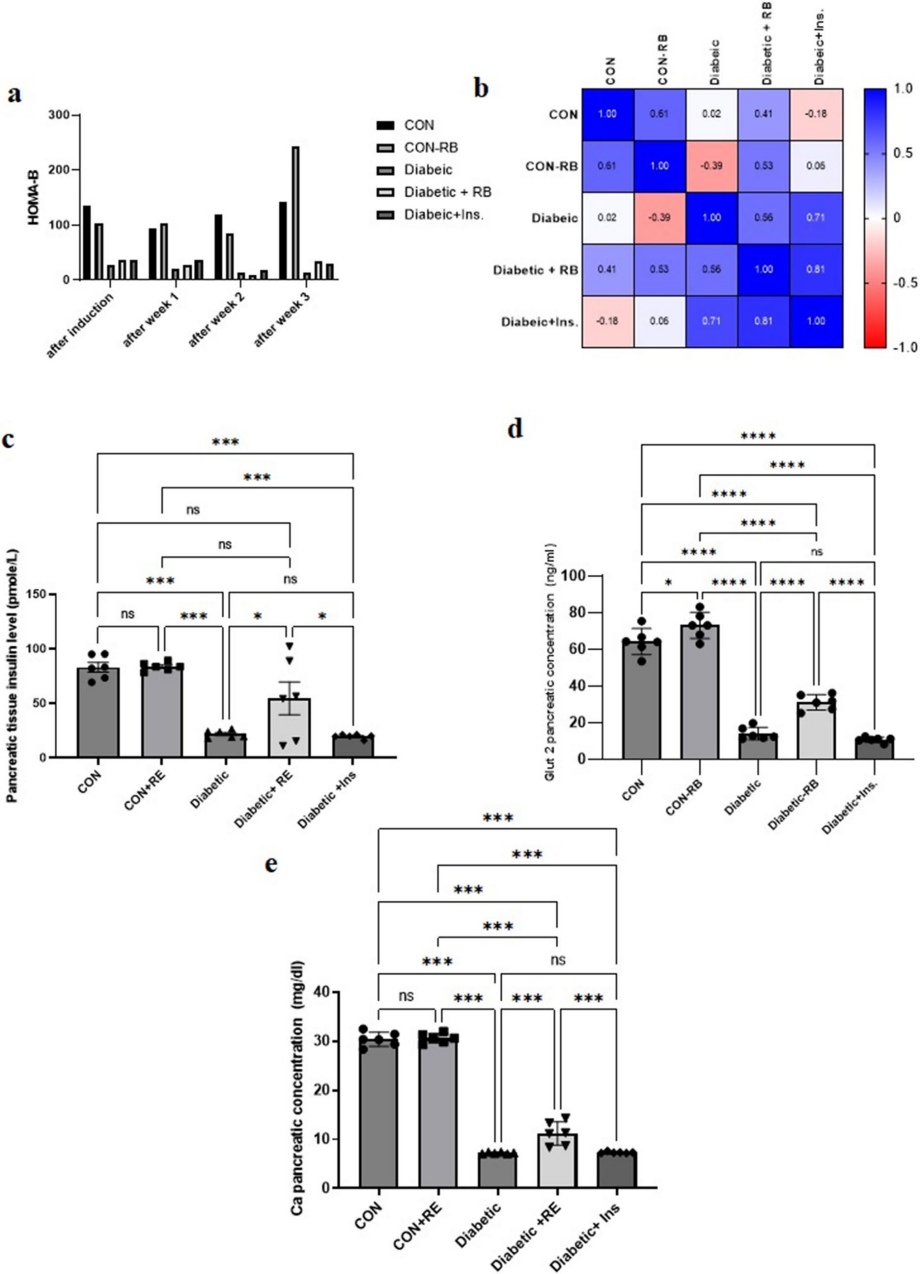
**Effect of daily oral treatment with RB (100 mg/kg/day) and Ins. (5 IU/200 g/day) on HOMA-β and pancreatic biochemical parameters:** Effect of treatment with RB (100 mg/kg/day, P.O.), and Ins. (5 IU/200 g/day, S.C**)**on HOMA-β (a, b) along weeks of the experiment and on pancreatic tissue levels for c) insulin d) GLUT2 e) calcium at the end of the experiment in STZ diabetic rats. Rats were injected with multiple low doses of STZ on the first four days HOMA-beta values calculated as:] blood insulin concentration (μU/ml) ×20[/] blood glucose (mmol/L) -3.5[ (**a**): Statistical analysis was done using two-way ANOVA followed by a Bonferroni post-test (**b**): Pearson correlation map for HOMA-beta values. The correlation coefficient is shown by the color bar: dark blue indicates strongly positive correlations, light blue indicates week positive correlation, white denotes negative correlations, and red denotes strongly negative correlations (**c**), (**d**), (**e**): Statistical analysis was done using One-way ANOVA followed by a Tukey post-test. Values are presented as mean ± SEM (*n*=6)

In Fig. 3a, all diabetic rats, after induction, displayed a significant decline in β-cell capacity in insulin secretion compared to control groups. However, in both diabetic-treated groups there was a continuous decline through weeks 1 and 2, whereas at week 3, the RB diabetic-treated group showed a numerically insignificant increase in the HOMA-β index compared to the diabetic group. Interestingly, the CON + RB group showed a higher, non-significant increase in the HOMA-β index compared to the CON group at the end of the experiment. Using a correlation matrix in Fig. 3b, we could evaluate the RB-treated diabetic group strong relationship with CON and CON-RB groups (*P* = 0.592, 0.472 respectively), with a weaker relationship in relevance to the untreated diabetic group (*P* = 0.441), in contrast with the diabetic INS-treated group that holds a week relationship with CON and CON-RB groups (*P* = 0.821, 0.935 respectively) and a stronger relationship in relevance to the untreated diabetic group (*P* = 0.285). Where the *P*-value represents the probability of finding the correlation coefficient’s value indicating a greater correlation degree, given that the two variables are not actually correlated (null hypothesis).

#### Pancreatic insulin level in STZ diabetic rat

As shown in Fig. 3c, rats'pancreatic insulin levels vary between different groups (F (25, 4) = 19.47). Results presented a significant decline in pancreatic insulin levels in the diabetic group by 73.57 (*P* < 0.001) compared to the CON group and 73.77% (*P* < 0.001) compared to the CON + RB group. INS-treated diabetic group insulin level decreased with no significant difference from the diabetic rats. While surprisingly, in the RB-treated diabetic group, insulin levels returned to normal levels, with a significant difference from the diabetic rats by 149.13%. (*P* = 0.024).

#### Pancreatic Glut2 level in STZ diabetic rat

Figure 3d reflects the variation in rats'pancreatic Glut2 levels between different groups (F (25, 4) = 251.8, *P* < 0.001). The diabetic group displayed a significant decrease in pancreatic Glut2 level from the CON group by 80.937% and from the CON + RB group by 80.887%. When compared to the diabetic group, the diabetic INS-treated group's pancreatic Glut2 level remained unchanged. In contrast, the diabetic RB-treated group's pancreatic Glut2 levels increased significantly by 126.495%. Noteworthy, there is still a significant difference between diabetic RB-treated group pancreatic Glut2 levels related to the CON group. Remarkably, in the CON + RB group, pancreatic Glut2 levels increased significantly compared to the CON group.

#### Pancreatic calcium levels in STZ diabetic rat

The disparity in pancreatic calcium levels among groups is revealed in Fig. 3e (F (25, 4) = 498.2, *P* < 0.001). Diabetic group pancreatic calcium level significantly dropped by 76.168% from CON and 76.339% from CON + RB. The INS-treated group's pancreatic calcium levels do not differ from those of the diabetic group. In contrast, the diabetic group treated with RB had a significant increase in pancreatic calcium levels of 54.589% when compared to the diabetic group. However, it is important to note that the pancreatic calcium level of the diabetic RB-treated group differs significantly from that of the CON group.

Effect of Rice bran extract and Insulin on pancreatic mRNA expression level of PPARℽ, SERCA, PrKc, and PDX1 genes involved in the regulation of insulin secretion in STZ diabetic rats.

#### PPARℽ expression levels

**Fig. 4:**
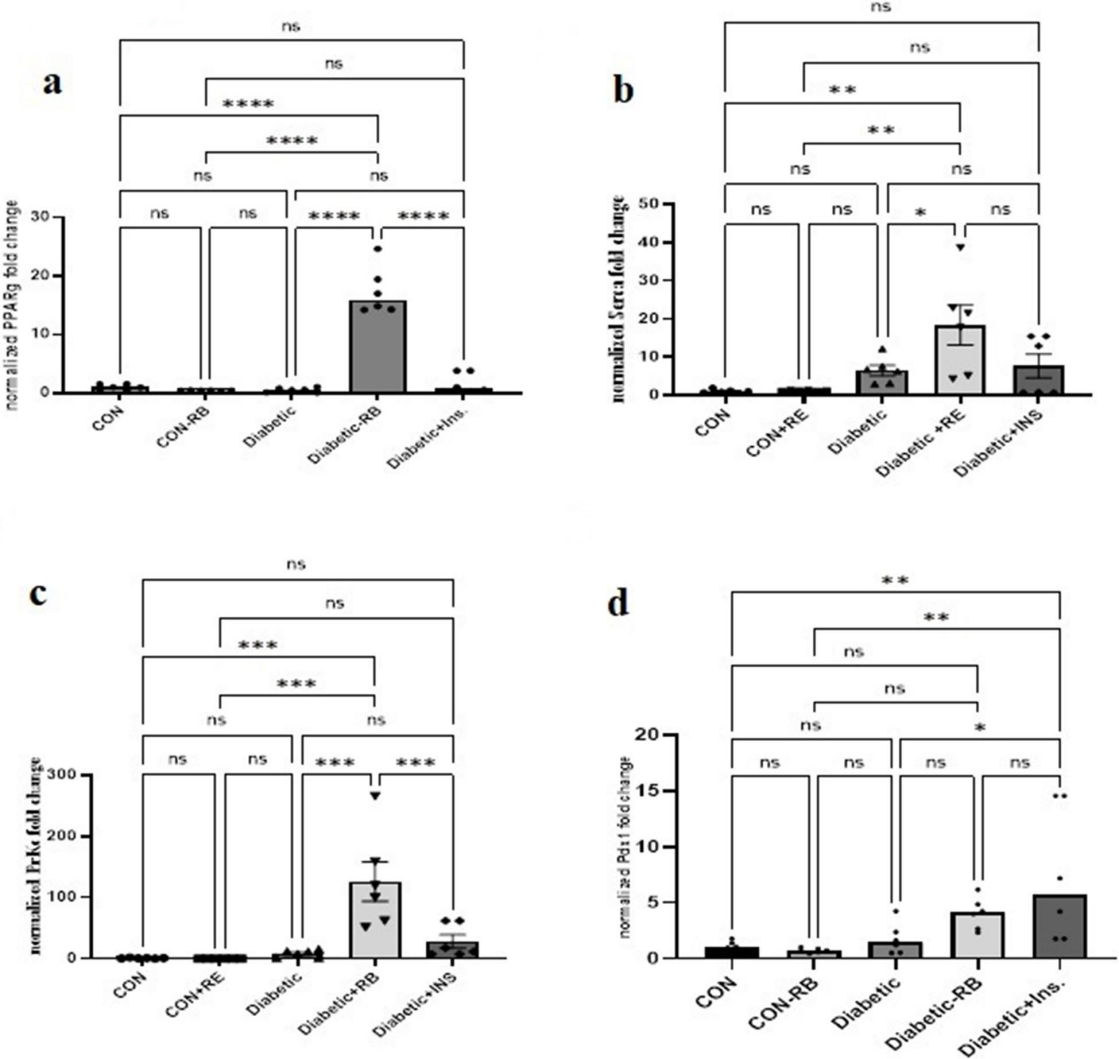
**Effect of oral treatment with RB (100 mg/kg/day), and Ins. (5 IU/200 g/day) on pancreatic gene Expression:** Effect of treatment with RB (100 mg/kg/day, P.O), and Ins. (5 IU/200 g/day, S.C)on pancreatic gene Expression Levels for **a**) PPARℽ **b**) SERCA **c**) Prkc **d**) Pdx1 at the end of the experiment in in STZ diabetic rats. Rats were injected with multiple low doses of STZ on the first four days Statistical analysis was done using One-way ANOVA followed by a Tukey post-test Values are presented as mean ± SEM (*n*=6)

As shown in Fig. 4a, unexpectedly, the PPARℽ expression level in the diabetic group slightly decreased from the control groups (CON and CON + RB) with no significant difference. The INS-treated group showed no significant difference between the related control groups and the diabetic group. On the contrary, the RB-treated diabetic group demonstrated significant up-regulation (F (25, 4) = 83.09, *P* < 0.0001) in PPARℽ expression levels higher than the CON group by 1646.1% and higher than the diabetic group by 3303.797%. Also, there is no significant difference between the insulin-treated group related to the CON group or the diabetic group.

#### SERCA expression levels

Figure 4b showed a minor increase in SERCA expression levels in diabetic rats with no significant difference compared to control groups (CON and CON + RB). In addition, SERCA expression level up-regulation in the INS-treated diabetic group has no significance related to the diabetic group or control group. While SERCA Expression level significantly up-regulated in RB-diabetic-treated rats 3 times compared to the diabetic group, and 18 times compared to the CON group (F (25, 4) = 6.42, *P* < 0.05).

#### Prkc expression levels

It was noticed in Fig. 4C a slight rise in Prkc expression levels in the diabetic group with no significant difference related to the control group. As well, in the INS-treated diabetic group, Prkc expression level up-regulation showed no significant change compared to the CON and diabetic groups. However, in the RB-treated diabetic group, significant up-regulation in Prkc expression levels was 15 times compared to the diabetic group and 126 times compared to the CON group (F (25, 4) = 12.40, *P* < 0.05).

#### PDX1 expression levels

Figure 4d presented an unanticipated, non-significant rise in PDX1 expression levels in diabetic rats related to the control group. INS-treated rats revealed significant PDX1 expression up-regulation (F (25, 4) = 5.824, *P* < 0.005) by 316.262% related to the diabetic group. Rather unexpectedly, in the RB-treated diabetic group, PDX1 expression levels displayed no significant difference compared to the diabetic group.

### Effect of rice bran extract and insulin on STZ-induced histological alteration in STZ diabetic rat

#### Pancreatic tissue (H&E stain)

**Fig. 5:**
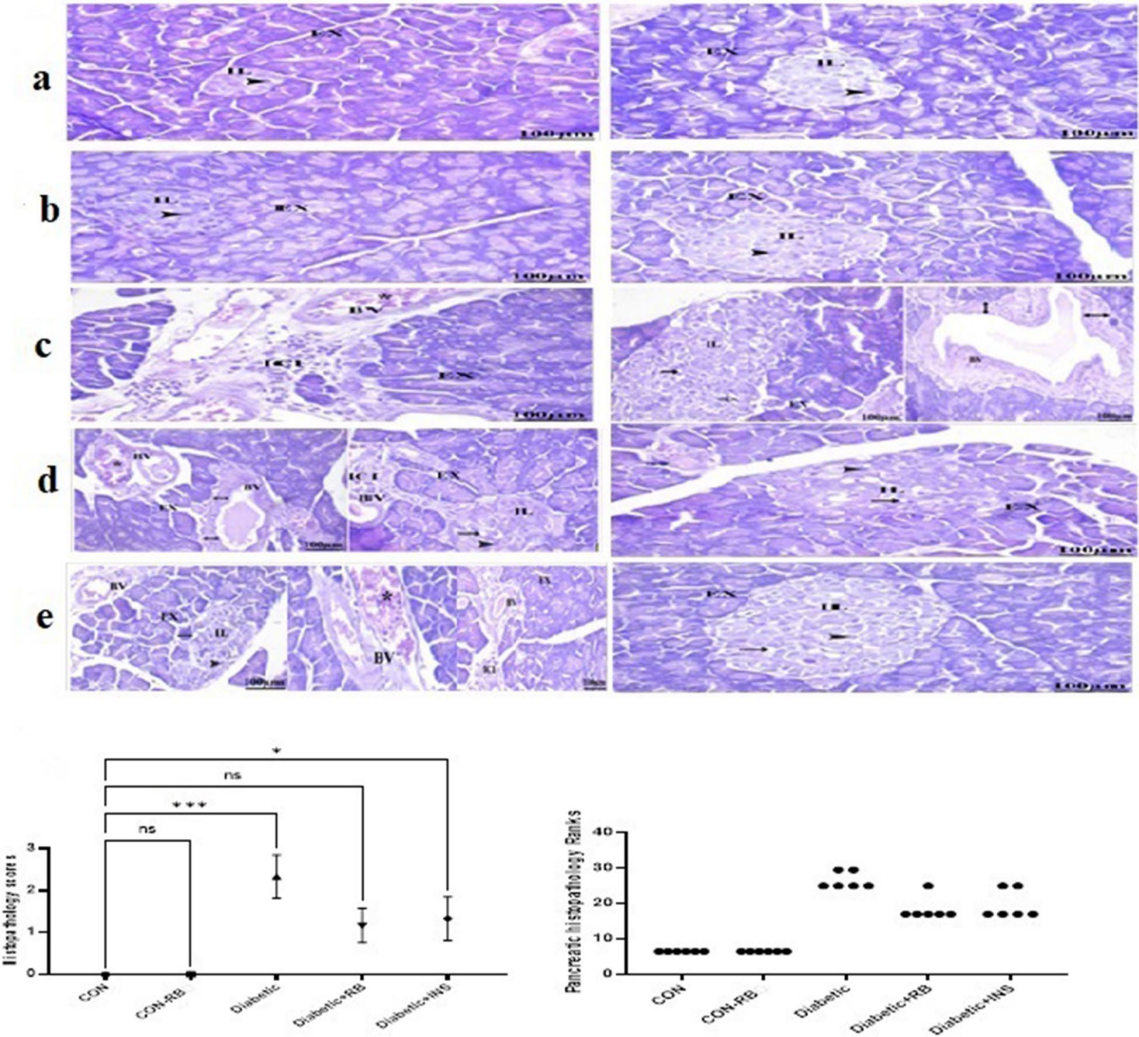
**Effect of oral treatment with RB (100 mg/kg/day), and Ins. (5 IU/200 g/day) on pancreas histopathology: ** Effect of treatment with RB (100 mg/kg/day, P.O.), and Ins. **(5 IU/200 g/day, S.C.) **on pancreas histopathology in STZ- diabetic rats Photomicrographs of rat pancreas sections stained with H&E (X100), **a**) control rats **b**) RB- control treated rats, **c**) Diabetic rats **d**) RB-treated Diabetic rats **e**) INS- treated diabetic rats As shown, the control groups, the pancreas exhibited a normal histopathological structure. The pancreas in STZ-diabetic rats showed exocrine acini (EX) cell atrophy, vacuolation (arrow), necrosis, and a significant decline in the number of beta cells leading to the islets of Langerhans (IL), dilated (double head arrow), congested (*) blood arteries and mononuclear cells (ICI) infiltrated. RB-treated diabetic rats had apparently mild vacuolation (arrow), congested (*) blood arteries and mononuclear cells (ICI) infiltrated. As well as a moderate increase in Islets of Langerhans (IL). The pancreas of INS-treated diabetic rats displayed moderate vacuolation (arrow), congested (*) blood arteries and mononuclear cells (ICI) infiltrated. As well as a moderate increase in Islets of Langerhans (IL). Statistical analysis was done using the Kruskal-Wallis test followed by a Dunn’s post-test. Values are presented as mean ± SEM

In Fig. 5, the CON group displayed normal pancreatic architecture. The acinar cells that make up the exocrine (EX) portion of the pancreas are arranged into tiny lobules and tightly packed. The CON + RB group revealed the pancreas'distinct lobules and external secretory units involving several pyramidal cells. Furthermore, internal secretory units (IL) represented several hormone-secreting cells next to the blood vessel.

In diabetic rats, the overall structure is deformed. Most exocrine acini displayed cell atrophy as a sign of acinar damage. Acini demonstrated a degree of decline. Degeneration, vacuolation, necrosis, and a significant decline in the number of beta cells lead the islets of Langerhans (IL) to decrease. Most of the areas showed thickly walled, dilated, and congested blood arteries.

In Both treated groups Mononuclear cells (ICI) infiltrated and clogged up some blood vessels (BV) still present, however, both showed some progress whereas vacuolation is mild in both groups. Besides, in insulin-treated rats, minor acinar cell aging was exhibited. As well, it is worth taking a look at RB-treated rats'pancreas which revealed slight improvement in which Some IL inside the acinar cells has several beta cells and a regular shape and size.

#### Brain tissue (Congo red stain)

**Fig. 6:**
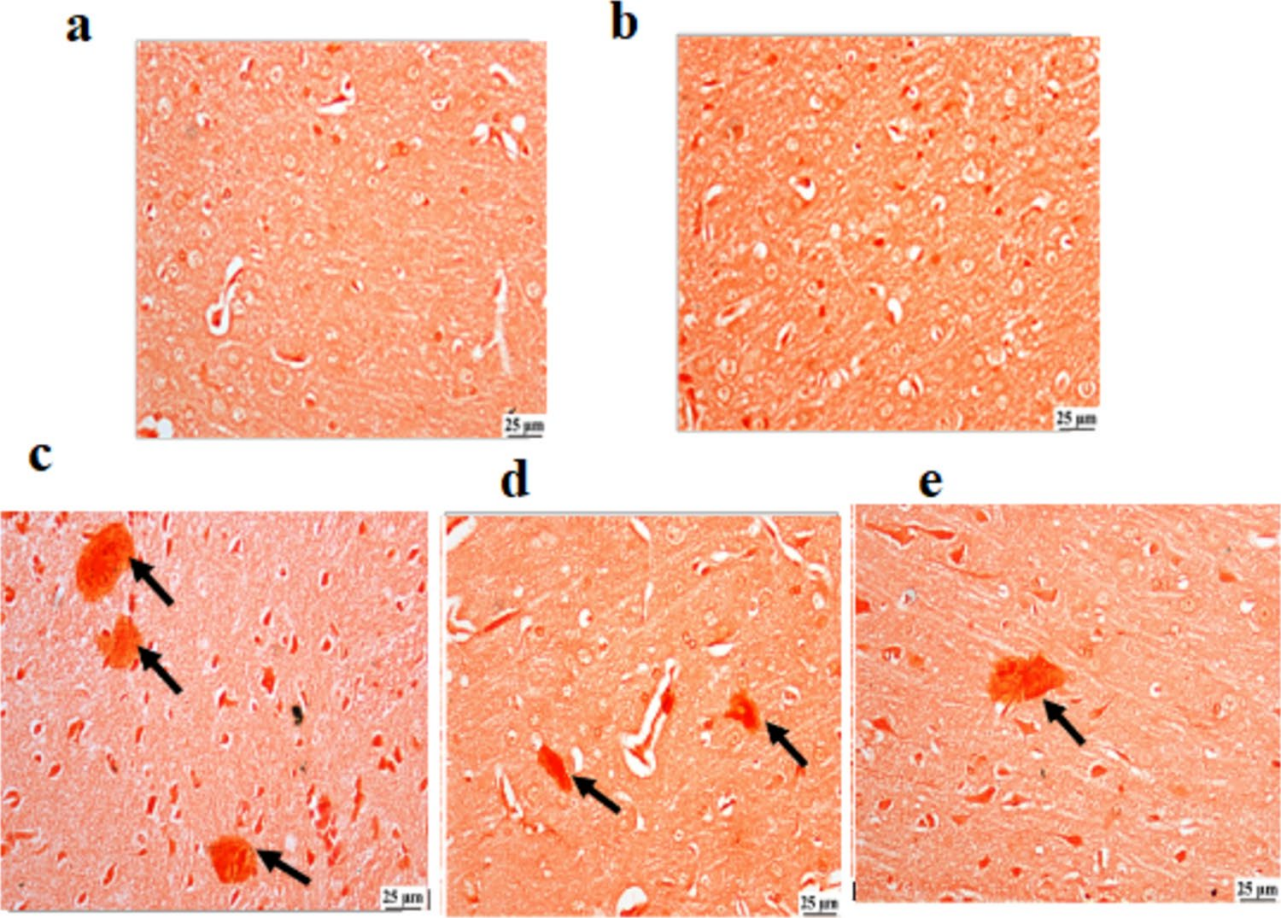
**Effect of treatment with RB (100 mg/kg/day) and Ins. (5 IU/200 g/day) on Amyloid plaque formation:** Photomicrographs of rat brain sections stained with Congo red (X25), **a**) control rats, **b**) RB-control-treated rats, **c**) Diabetic rats, **d**) RB-treated Diabetic rats, **e**) INS-treated diabetic rats Control groups showed normal staining with Congo red. Diabetic group presented numerous dark red-stained plaques (arrows). INS-treated diabetic rats revealed sporadic red-stained plaque (arrow). RB-treated diabetic rats had few red-stained plaques (arrows)

Histopathological findings presented in Fig. 6 were based on descriptive observations without quantitative scoring or statistical analysis. This lack of quantitative analysis constitutes a limitation and should be considered when interpreting the results. Congo red staining of brain sections from the CON group and CON + RB group revealed normal faint staining with the absence of any dark-stained plaques. On the contrary, the diabetic group showed numerous red-stained plaques scattered within the brain tissue. The insulin-treated rats showed sporadic red-stained plaques in a few sections, whereas the RB-treated rats showed fewer red-stained plaques.

## Effect of rice bran extract and insulin on rats'behavioral alterations

### Novel object recognition test in STZ-diabetic rats

#### Y-maze test in STZ-diabetic rats

**Fig. 7:**
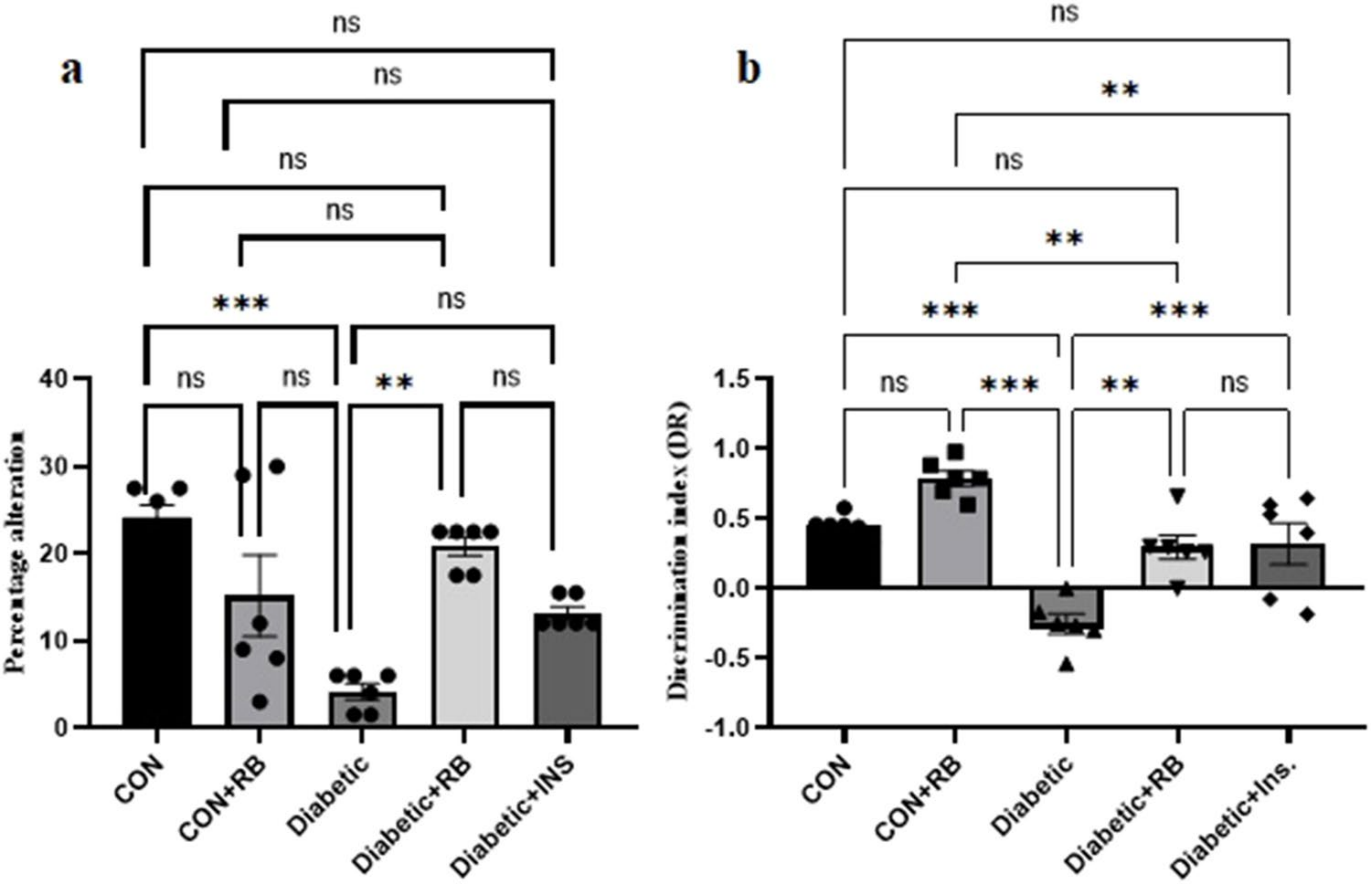
**Effect of oral treatment with RB (100 mg/kg/day) and Ins. (5 IU/200 g/day) on behavioral tests:** Effect of treatment with RB (100 mg/kg/day, P.O.) and Ins. (5 IU/200 g/day, S.C.)on (**a**) percentage alteration in the Y-maze test and (**b**) discrimination index in the Novel Object Recognition (NOR) test at the end of the experiment in STZ diabetic rats. Rats were injected with multiple low doses of STZ on the first four days For NOR test statistical analysis was done using one-way ANOVA followed by a Tukey post-test. For Y-maze test statistical analysis was done using Kruskal-Wallis followed by a Dunn’s post-test. Values are presented as mean ± SEM (*n* = 6)

As displayed in Fig. 7a, rats’ spatial memory performance differs significantly within the 5 groups (*P* = 0.0009). The diabetic group showed an inability to opt for the correct choice, showing a significant decrease in the percentage of alteration compared to the CON group by 56.99%. RBE and INS-treated groups improved rats'spatial memory, however, only RBE-treated group displayed a significant increase in the percentage of alteration by 123.66% from the diabetic group. Remarkably, both treated groups revealed no significant difference in the percentage of alteration from the normal CON group.

#### Novel object recognition test in STZ-diabetic rats

Figure 7b demonstrates rats'recognition behavior altered among groups (F (25, 4) = 18.64). Results showed a significant decline in the discrimination ratio in the diabetic group (156%, *P* < 0. 001) compared to CON. Diabetic groups treated with RB and INS restored the ability to explore new objects and showed a significant increase by 217% (*P* = 0. 001) and 226% (*P* < 0.001), respectively, from the diabetic group. Remarkably, both treated groups revealed no significant difference in discrimination ratio from the normal CON group.

#### Novel object recognition test in STZ-diabetic rats

Figure 7b demonstrates rats'recognition behavior altered among groups (F (25, 4) = 18.64). Results showed a significant decline in the discrimination ratio in the diabetic group (156%, P < 0. 001) compared to CON. Diabetic groups treated with RB and INS restored the ability to explore new objects and showed a significant increase by 217% (*P* = 0. 001) and 226% (*P* < 0.001), respectively, from the diabetic group. Remarkably, both treated groups revealed no significant difference in discrimination ratio from the normal CON group.

## Discussion

In the present experiment, administration of STZ for four sequential days induced T1D, which displayed an acute rise in blood glucose (Fig. 1a), reflecting a significant drop in β-cell insulin secretory function as indicated by the HOMA-β index (Figs. 3 a, b and c). With sustained hyperglycemia (Fig. 1) at the end of the experiment, the diabetic group showed a decline in pancreatic Ca^2+^ levels as a result of the decrease in pancreatic GLUT2 levels (Fig. 3 c, d and e). The reduction in pancreatic GLUT2 levels is consistent with published research, which links GLUT2 expression decline to an increase in free fatty acids and triglycerides in the blood as a result of β-cell dysfunction (Thorens 2001). GLUT2 levels decline, reducing glucose uptake by β-cells and impairing glucose-6-phosphate phosphorylation and the production of ATP in the glycolysis process. This hinders glucose-inducing intracellular ATP levels from increasing the cytoplasmic ATP/ADP ratio for K + -ATP channel shutting and voltage-dependent Ca^2+^ channel opening. This reduces Ca^2+^ release, ultimately leading to inhibition of exocytotic insulin release from insulin-containing granules (Sun et al. 2023). The observed fall in pancreatic GLUT2 and Ca^2+^ levels in the diabetic group was also reflected in a drop-in blood insulin levels (Klec et al. 2019; Trexler and Taraska 2017), highlighting the role of glucose transport in β-cells in glucose-stimulating insulin secretion (GSIS) (Saji et al. 2020).

RB treatment had an inverse effect on insulin levels, showing a significant increase in blood insulin levels compared to the insulin-treated group, as shown at the end of the experiment (Fig. 2d). Moreover, in pancreatic tissue, only RBE significantly increased pancreatic insulin levels compared to untreated and insulin-treated groups (*p* < 0.001, *p* < 0. 1) (Fig. 5A). This effect was corroborated by the strong correlation of the HOMA-β index between the RB-treated group and control groups rather than with the insulin-treated group (Fig. 3a and b). On the contrary, for blood glucose levels, only insulin therapy showed a significant reduction in diabetic rats, while RBE showed a slow, non-significant decline until the end of the experiment. Interestingly, the same finding regarding blood glucose levels was reported by Kaup et al. (2012) when examining the RBE administration for glucose-treated rats (2 g/kg BW) and abruptly elevated plasma insulin. The author argued that the organism requires tight glucose regulation in the long term and suggested prolonged RBE treatment to adjust blood glucose levels. This assumption is supported by the reported effect of tocotrienol, the rice bran oil fraction, on reducing fasting blood glucose and HbA1 C in a T1D rat model after 8 weeks of diabetes induction (Siddiqui et al. 2010). It is well known that the antidiabetic effect of tocotrienols is a long-term PPAR-mediated activity (Fang et al. 2010). Like other stabilized RB supplements, they reduced HbA1 C in T2D patients after 12 weeks (Cheng et al. 2010).

Diabetic rats treated with RBE showed a significant rise in pancreatic GLUT2 levels compared to those treated with insulin (Fig. 3d). This is the first study to report such an effect of RBE on GLUT2 levels in the pancreas. Nevertheless, it's worth noting that RBE vitamin E components, tocotrienols (δ- and γ-T3), have a dose-dependent effect on increasing GLUT2 expression in rat pancreatic islets (Chia et al. 2016). The RBE control group showed an intriguingly significant increase in GLUT2 levels compared to the untreated control group (Fig. 3d), suggesting a possible effect of RBE on GLUT2 basal levels at normal conditions—an observation that needs further investigation.

This, in turn, was consistent with a significant increase in intracellular Ca^2+^ ion levels in the RBE-treated group compared to insulin (Fig. 3e). This infers an increase in glucose transport inside β-cells (Saji et al. 2020), leading to an increase in Ca^2+^ ion influx through ATP-induced Ca^2+^ channel opening (Sun et al. 2023). Vanillic acid, one of the phenolic compounds of the RBE phenolic fraction (supplementary data S2-S5), has been reported to increase Ca^2+^ influx through a voltage-dependent calcium channel (Mahendra et al. 2019). Additionally, another phenolic compound, ferulic acid, a main active metabolite of RBE γ-oryzanol (Arumsari et al. 2019; Kokumai et al. 2019), stimulated Ca^2+^ influx in INS-1 cells, supporting RBE active components in their role in GSIS (Ruamyod et al. 2023). On the contrary, levels of pancreatic GLUT2 and Ca^2+^ were not altered in the insulin-treated group in relation to the untreated diabetic group (Fig. 3d and e). This indicates no impact of insulin treatment on GSIS, in agreement with Persaud et al.'s (2001), where the study disclosed the insulin-inhibitory effect of its own secretion in human islet cells. Other insulin therapy studies showed an acute decrease in β-cell GLUT2 in STZ-induced diabetic rats (Thulesen et al. 1997).

To reveal the molecular mechanism underlying the influence of RBE administration on insulin secretion signaling pathways, we analyzed the gene expression levels of PPARγ, SERCA, PKC, and PDX1. Interestingly, RBE showed a highly significant increase in PPARγ pancreatic gene expression in comparison to the untreated diabetic group (Fig. 4a, *p* < 0.0001). This finding supports our previously reported RBE-PPARγ agonist activity (Abd El Fattah et al. 2020; El-Din et al. 2021; Mostafa et al. 2018). The potential of RBE in regulating molecules in the insulin signaling cascade is also evidenced by the reported effect of tocotrienols on increasing PPARγ expression in glucose-stimulated pancreatic β-islet cells (Chia et al. 2016). On the contrary, although insulin is a PPARγ receptor regulator (Rieusset et al. 1999), insulin treatment in the present study showed an insignificant effect on PPARγ gene expression compared to the untreated diabetic group. The precise impact of insulin on PPARγ expression is not well comprehended. However, the ERK proteins and activated signaling molecules provided a plausible explanation for PPARγ-insulin interaction through ERK-1/2-Junk phosphorylation of the PPARγ-insulin signaling cascade in adipose tissue. Studies have found that PPARγ phosphorylation decreased PPARγ ligand's transcriptional activity as proteasomal degradation increased, leading to the suppression of PPARγ expression (Leonardini et al. 2009; Floyd and Stephens 2002).

RBE administration resulted in a significant increase in SERCA2 gene expression (Fig. 4b), surpassing both diabetic and control groups. This finding supports the link between PPARγ and SERCA2 gene expression, which is regulated by PPARγ interaction with the PPAR-responsive element in the human SERCA2 promoter (Kono et al. 2012). The increase in SERCA2 expression aligns with the observed rise in Ca^2+^ levels (Fig. 3e) in the RBE-treated group, indicating that SERCA2 maintains high intracellular Ca^2+^ levels (Trexler and Taraska 2017). The use of RBE resulted in a notable increase in PrkC levels in the diabetes-treated groups compared to insulin (*p* > 0.001) (Fig. 4c). This finding suggests that the rise in Ca^2+^ influx activates PrKC βII (Shimabukuro et al. 1998), which in turn stimulates Na + -permeable channels, leading to membrane depolarization and insulin secretion (Fleming and Storz 2017). Moreover, the increase in PrKC βII is expected to redistribute GLUT2 to transport glucose molecules inside the β-cell (Cohen et al. 2014). This condition was obviously detected in our current results, as illustrated before (Fig. 3d). It is worth noting that ferulic acid can be reported to activate pathways like PKA, CaMKII, MAPK/ERK, and PI3 K to influence PrkC expression (Zeni et al. 2012), which may promote efficient GSIS. Although PDX1 gene expression increased in the RB-treated diabetic group compared to the untreated diabetic group, it was insignificant (Fig. 6D). RBE's insignificant effect on PDX1 for insulin gene expression could be explained by the possible direct up regulatory effect reported to some RBE active components (tocotrienols and phenolic fractions) on the INS-1 gene rather than PDX1 (Chia et al. 2016; Saji et al. 2020). Since this is the first study to examine the potential impact of RBE on insulin secretion in a TID model, more research is needed to clarify the precise mechanism for RBE on PDX1 gene expression. On the other hand, a significant increase in PDX1 was observed in the insulin-treated group as compared to the diabetes-untreated group. This increase occurs via PDX1 phosphorylation and translocation to the nucleus, as attributed to Rachdaoui, considering the short-term autocrine effect of insulin in human islet cells (Rachdaoui 2020).

RBE Improving cognitive abilities was examined by evaluating the performance of diabetic rats in Y-maze and NOR behavioral tests. The findings of RBE administration demonstrated a significant improvement in cognitive functions, with no notable disparity observed between the treated groups (Fig. 7a and b) compared to the diabetic group. The outcomes align with our earlier findings that Egyptian RBE enhances rats’ cognitive function in an LPS-induced neuroinflammatory animal model (Mostafa et al. 2018). In a corresponding manner, Hagl et al. (2016) conducted a study that demonstrated the efficacy of RBE in restoring cognitive abilities in aged rats. This restoration was achieved through the activation of peroxisome proliferator-activated receptor gamma coactivator 1-alpha (PGC1α), which directly regulates PPARγ. Regarding insulin treatment, Strachan's (2005) findings reported the effectiveness of intranasal insulin therapy in improving declarative memory. Insulin, whether generated internally or externally, has been found to potentially promote synaptic remodeling and synaptogenesis, which are crucial for memory consolidation (Cholerton et al. 2013; Skeberdis et al. 2001). Additionally, it induces the expression of neurotransmitters like acetylcholine and norepinephrine, regulating cognitive functions through the coordination of cholinergic and adrenergic pathways (Cholerton et al. 2013; De Leo et al. 2023). Thus, the study suggests that RBE may enhance memory and cognition by increasing peripheral insulin secretion through PPARγ regulator activity, potentially acting in the central nervous system. Furthermore, Congo-red staining brain sections showed fewer red Aβ plaques in the brain tissue of the treated groups compared to untreated diabetic rats (Fig. 7). This observation indicates a decline in the formation of amyloid plaques, as demonstrated in our previous study on the neuroprotective mechanism of RBE in mice with LPS-induced neuroinflammation. (Abd El Fattah et al. 2020). Insulin impacts tau phosphorylation and amyloid-β peptide clearance, which are Alzheimer's disease pathological features (Boccardi et al. 2019). This lends credence to the association between diabetes and dementia. Histological examination results supported the RBE effect on restoring pancreatic structure more effectively than insulin treatment, as shown in Fig. 5. Untreated diabetics showed distortion in pancreatic structure, atrophy in exocrine glands, and decreased β-cells and IL numbers, which corresponds to the acute rise in blood glucose (Fig. 1). Interestingly, rats treated with RBE showed several normal β-cells with regular shape and size, disclosing upgraded IL structure inside the acinar cell compared to insulin treatment. A result in alignment with the reported effect of red RBE on enhancing pancreatic structure in n mice treated with a high-fat diet (Munkong et al. 2022) and reinforced by the role of pioglitazone PPARγ agonistic effect in shielding pancreatic β-cells from the effects of hyperglycemia, deficiencies in insulin secretion, and the impact of cell death. This protective mechanism is achieved by activation of the SERCA2b pump via direct enhancement of; SERCA2b gene expression. This supports our present results on the effect of RBE on SERCA2b gene expression.

## Conclusion

In conclusion, our RBE treatment enhanced insulin secretion, obviously with little influence on glucose levels, which we suggest it should be reversed by longer-term supplementation. This finding suggests that RBE's beneficial effect on cognitive performance may be related to an increase in insulin levels in the brain. The RBE effect on enhancing β cell insulin secretion provides further insights into the RBE mechanism of action by targeting the PPARγ/PDX1 signaling pathway. This is evidence-based action by RBE components.

## Supplementary Information

Below is the link to the electronic supplementary material.

Supplementary file1 (DOCX 780 KB)

## Data Availability

No datasets were generated or analysed during the current study.

## Abbreviations

T1D: Type 1 diabetes
PPAR-γ: Peroxisome proliferator-activated receptor
TZDs: Thiazolidinedione
GSIS: Glucose-stimulating insulin secretion
PDX1: Pancreatic and duodenal homeobox 1
GLUT2: Glucose transport 2
SERCA: Sarcoendoplasmic reticulum calcium ATPase
PrKC: Protein Kinase C
PDX1: Pancreatic and duodenal homeobox 1
DCVs: Dense core vesicles
RBE: Rice bran Extract
PGC1α: Proliferator-activated receptor gamma coactivator 1-alpha
