# Supplementary material for: Rice bran extract attenuates cognitive impairment by enhancing pancreatic β-cell insulin secretion in STZ-induced diabetic rats targeting the PPARγ/PDX1 pathway
Source: Metab Brain Dis. 2025 Jun 19;40(6):228. doi: 10.1007/s11011-025-01639-1 (PMC12178994; doi:10.1007/s11011-025-01639-1)
Supplement: Supplementary file 1 — Supplementary file1 (DOCX 780 KB) [file 11011_2025_1639_MOESM1_ESM.docx]

**Metabolic brain disease Journal**

**Rice Bran Extract attenuates cognitive impairment by enhancing pancreatic β-cell insulin secretion in STZ-induced diabetic rats targeting the PPARγ/PDX1 pathway**

**Madonna M. Youssef ^1^, M.F. El-Yamany ^2^, Reham M. Abdelkader^3^, Ola A. Heikal ^4^**

1. Pharmacovigilance Department, Egyptian Drug Authority, Giza, Egypt. Email: [Madonna.magdy@std.pharma.cu.edu.eg](mailto:Madonna.magdy@std.pharma.cu.edu.eg) (**Corresponding Author**). Orcid ID:0000-0002-0186-7242
2. Pharmacology & Toxicology Department, Faculty of Pharmacy, Cairo University, Cairo, Egypt. Email: [Mohammed.elyamany@pharma.cu.edu.eg](mailto:Mohammed.elyamany@pharma.cu.edu.eg) Orcid ID: 0009-0005-4970-7896
3. Pharmacology & Toxicology Department, Faculty of Pharmacy, German University, Cairo, Egypt. Email: [Reham.abdelkader@guc.edu.eg](mailto:Reham.abdelkader@guc.edu.eg) Orcid ID:0000-0003-3584-0045
4. Narcotics, Ergogenics & Toxins Department, National Research Center, Giza, Egypt. Email: [Dr.olaheikal@hotmail.com](mailto:Dr.olaheikal@hotmail.com) Orcid ID: 0000-0003-3320-6884

**Supplementary material**

**S1:** The timeline for experimental animal induction, administration of drugs, and behavioral assessment.

**
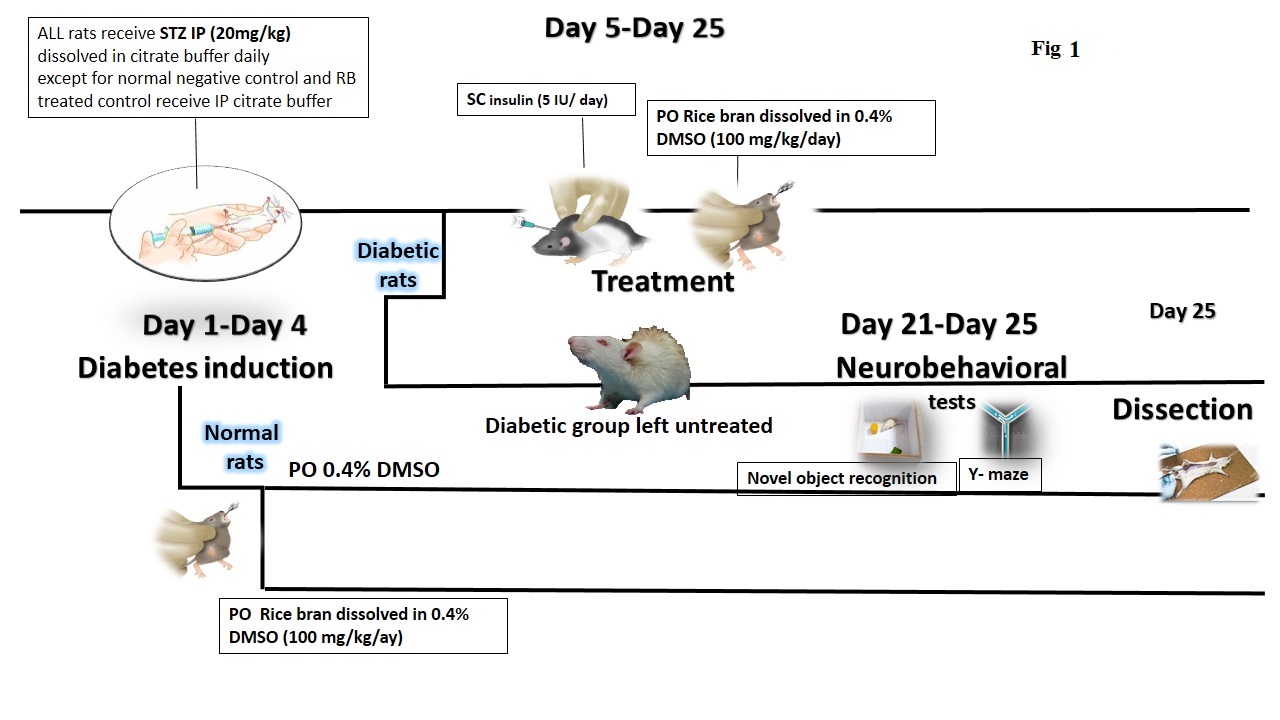
**

**S2: HPLC methodology for analysis** **of γ-oryzanol**

The bioactive compound of RBE, γ-oryzanol, was estimated using the reported HPLC method **(El-Askary, Hesham, 2012)** with some modifications. HPLC equipment used was Dionex Ultimate 3000 (Thermo Fischer). The separation was performed using a reversed phase column (Poroshell 120 EC-C18, 2.7 μm, 150 × 4.6 mm, Agilent, USA), provided with a C-18 guard column maintained at 21 °C. The elution using a linear isocratic program and methanol (100%) as a mobile phase, a flow rate of 1.2 ml/min, and an injection volume of 5μl with detection using a diode array detector at a wave length of 290 nm. were performed. The result calculation was done using Thermo Scientific Chromeleon software. Analysis of γ-Oryzanol content in the different RBE extracts was determined by comparison of retention time of γ-Oryzanol peaks in the different RBE extracts (i.e., RBE lab scale (H1; hexanoic & A1; alcohol), RBE industrial scale & enzymatic assisted oil G-RBO) with their respective peaks in γ-Oryzanol standard. and the γ-Oryzanol content was calculated using peak area measurement compared to their respective γ-Oryzanol standards, and the concentrations in the above RBE extracts were conducted from the constructed γ-Oryzanol calibration curve. All RBEs were stored in a refrigerator at 4 °C and warmed in a water bath at 37 °C just before use.

**

***HPLC chromatogram of* γ*- Oryzanol***

**S3: HPLC methodology for analysis** **of vitamins E congeners**

In the present report, measurement of the 4 natural vitamin E congeners α and γ Tocopherols and α and γ Tocotrienols in different prepared RBE oils was conducted. First calibration curves of 4 natural vitamin E congeners α and γ Tocopherols and α and γ Tocotrienols were constructed applying the reported HPLC method according to **Nadine *et al*., 2012,** with minor modifications. The HPLC equipment used was Dionex Ultimate 3000 from Thermo Fischer. The separation was performed using a reversed phase column (Phenomenex Kinetex PFP Column 2.6µm 150 x 4.6 mm), provided with a C-18 guard column maintained at 40 °C. The elution using a linear isocratic program and methanol: H2O (85:15) as a mobile phase, a flow rate of 0.8 ml/min, and an injection volume of 5μl-10μl, with detection using a diode array detector at a wave length of 290 nm, were performed. Peaks were recorded and integrated using Thermo Scientific Chromeleone software.


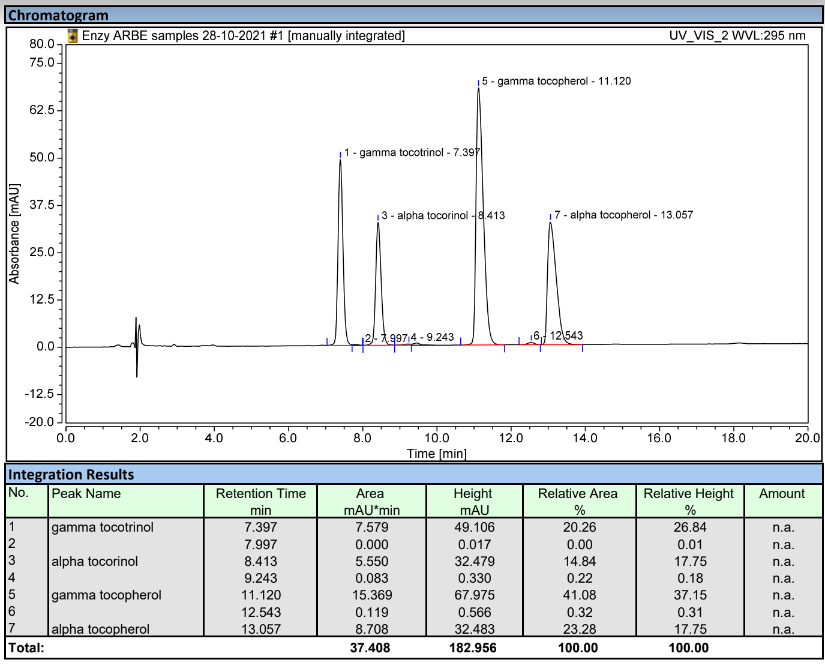


Figure S3: HPLC chromatogram of total Vitamin E congener’s standards

**Figure S3: Calibration curves of γ vitamin E isomers. γ and α Tocopherols and Tocotrienols**

**S4: Analysis of fatty acids profile of RBE A by GC/FID**

1 g **of RBE A** was analyzed for fatty acid profile using GC-FID under the following conditions.

**Gas chromatography**

The GC model 7890B from Agilent Technologies equipped with flame ionization detector at Central Laboratories Network, National Research Centre, and Cairo, Egypt was used. Separation was achieved using a Zebron ZB-FAME column (60 m x 0.25 mm internal diameter x 0.25 *μ*m film thickness). Analyses were carried out using hydrogen as the carrier gas at a flow rate of 1.8 ml/min at a split-1:50 mode, injection volume of 1 µl and the following temperature program: 100 °C for 3 min; rising at 2.5 °C /min to 240 °C and held for 10 min. The injector and detector (FID) were held at 250 °C and 285 °C, respectively.


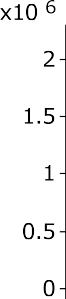

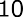

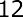

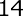

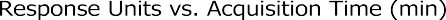

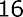

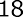

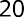

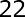

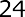

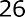

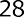

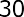

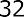

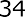

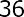

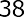

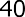

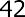

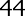

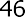

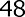

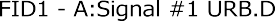


**Figure S4: GC chromatogram of Fatty acids profile**

**Table S4: Fatty acid profile of RBE**

| **RT** | **Name** | **Area** | **Area Sum %** |
| --- | --- | --- | --- |
| 18.812 | Myristic acid | 183950.92 | 0.38 |
| 24.738 | Palmitic acid | 8853815.8 | 18.27 |
| 25.737 | Palmitoleic acid | 82348.67 | 0.17 |
| 30.337 | Stearic acid | 995829.47 | 2.05 |
| 31.155 | Oleic acid | 19328050 | 39.88 |
| 32.785 | Linoleic acid | 17632243 | 36.38 |
| 34.717 | Linolenic acid | 647772.6 | 1.34 |
| 35.552 | Arachidic acid | 359583 | 0.74 |
| 36.16 | *cis*-11-Eicosenoic acid | 258886.45 | 0.53 |
| 40.539 | Behenic acid | 126706.9 | 0.26 |
| **RT** | **Name** | **Area** | **Area Sum %** |

### **S5 : LC/MS/MS**

**Conditions and parameters**

### **Instrument**

The analysis of the sample was performed using liquid chromatography–electrospray ionization–tandem mass spectrometry (LC-ESI-MS/MS) with an ExionLC AC system for separation and SCIEX Triple Quad 5500+ MS/MS system equipped with an electrospray ionization (ESI) for detection.

**Positive and negative MRM mode:**

The separation was performed using ZORBAX SB-C18 Column (4.6×100 mm, 1.8 µm). The mobile phases consisted of two eluents A: 0.1% formic acid in water; B: acetonitrile (LC grade)**.** The mobile phase was programmed as following, 2% B from 0-1 min, 2-60% B from 1-21 min, 60% B from 21-25 min, 2% B from 25.01-28 min. The flow rate was 0.8 ml/min and the injection volume was 3 µl. For MRM analysis of the selected polyphenols, positive and negative ionization modes were applied in the same run with the following parameters: curtain gas: 25 psi; Ion Spray voltage: 4500 and-4500 for positive and negative modes, respectively; source temperature: 400°C; ion source gas 1 & 2 were 55 psi with a declustering potential: 50; collision energy: 25; collision energy spread: 10.

**A) STD 1ppm**


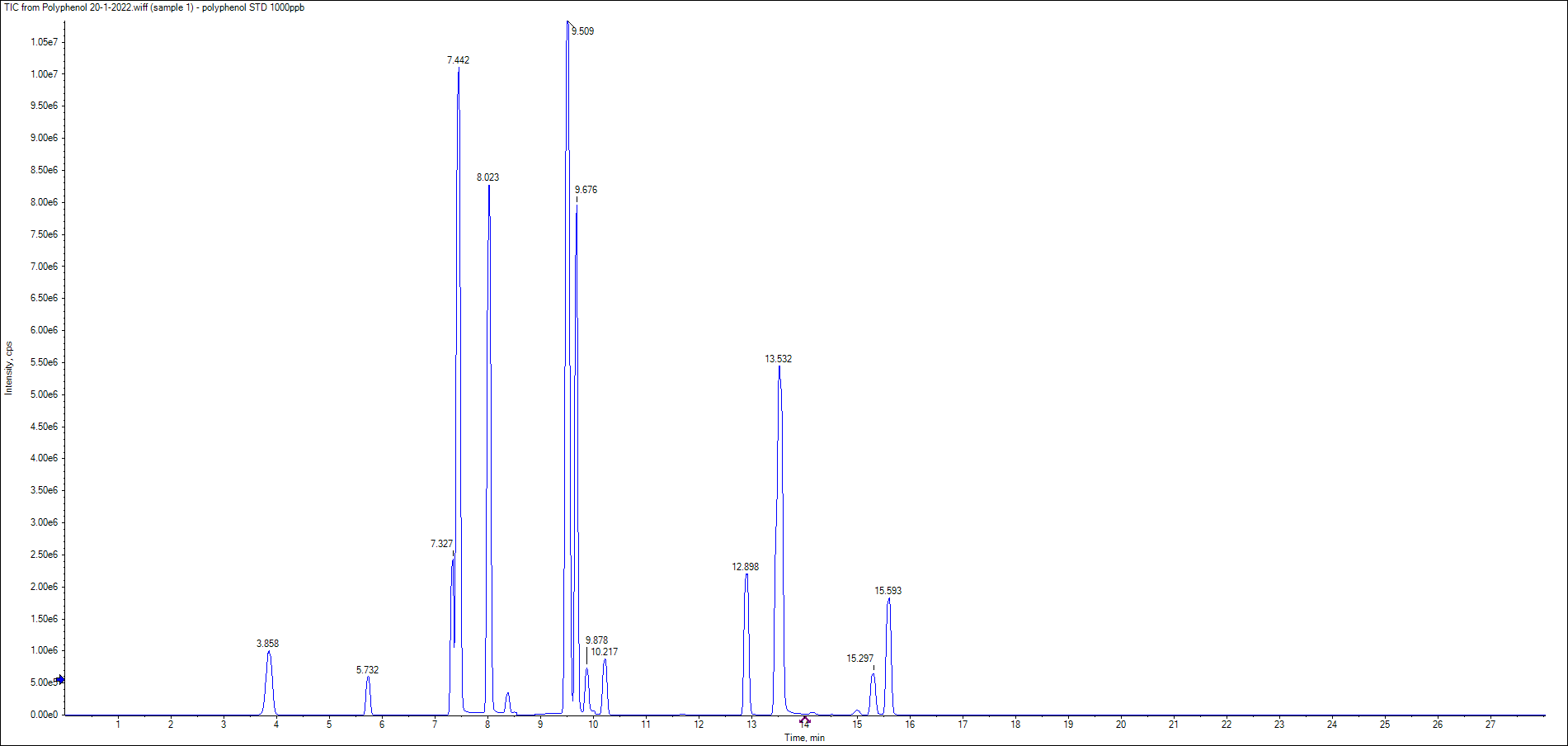


**B) RBE**


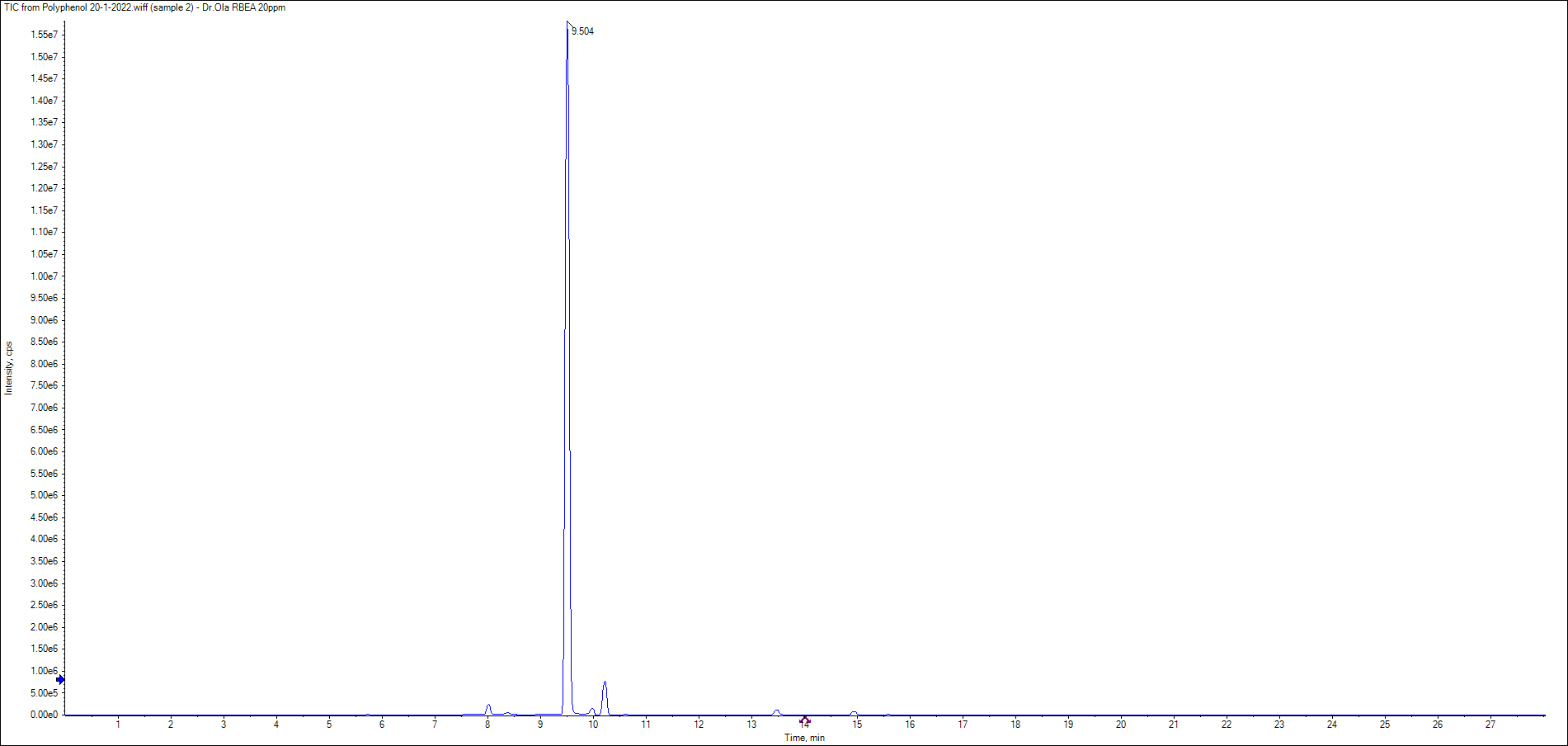


**Figure S5: LC/MS/MS of polyphenol standards; 1ppm (A) and RBE (B)**

**Table S5: Concentration of Phenolic compounds in Rice bran Extract**

| **Compound** | **Retention Time (RT)** | **Conc.in RBE in µg/gm** |
| --- | --- | --- |
| Chlorogenic acid | 7.34 | 0 |
| Daidzein | 12.88 | 0 |
| Gallic acid | 3.84 | 0.019831 |
| Caffeic acid | 8.03 | 0 |
| Rutin | 9.68 | 0.018559 |
| Coumaric acid | 9.52 | 69.53899 |
| Vanillin | 9.56 | 34.2467 |
| Naringenin | 14.95 | 64.29761 |
| Querectin | 13.55 | 0.208135 |
| Ellagic acid | 9.9 | 0.27467 |
| 3.4-Dihydroxybenzoic acid | 5.74 | 1.003323 |
| Hesperetin | 15.59 | 0.270073 |
| Myricetin | 11.86 | 0 |
| Cinnamic acid | 14.14 | 0 |
| Methyl gallate | 7.45 | 0.014511 |
| Kaempferol | 15.3 | 0 |
| Ferulic acid | 10.23 | 42.89549 |
| Syringic acid | 8.39 | 4.759162 |
| Apigenin | 15 | 1.465335 |
| Catechin | 7.32 | 0 |
| Luteolin | 13.48 | 1.853257 |

**Retention time of phenolic compounds and their concentration in µg/gm of RBE** **using LC/MS/MS chromatography analysis.**
